# Discrimination of Filter Coffee Extraction Methods of a Medium Roasted Specialty Coffee Based on Volatile Profiles and Sensorial Traits

**DOI:** 10.3390/foods12173199

**Published:** 2023-08-25

**Authors:** Agnese Santanatoglia, Laura Alessandroni, Lauro Fioretti, Gianni Sagratini, Sauro Vittori, Filippo Maggi, Giovanni Caprioli

**Affiliations:** 1 Chemistry Interdisciplinary Project (ChIP) Research Center, School of Pharmacy, University of Camerino, Via Madonna delle Carceri 9/B, 62032 Camerino, Italy; agnese.santanatoglia@unicam.it (A.S.); laura.alessandroni@unicam.it (L.A.); gianni.sagratini@unicam.it (G.S.); sauro.vittori@unicam.it (S.V.); giovanni.caprioli@unicam.it (G.C.); 2 Research and Innovation Coffee Hub, Via Emilio Betti 1, 62020 Belforte del Chienti, Italy

**Keywords:** coffee brewing methods, specialty coffee, Hario V60, French Press, Pure Brew, AeroPress, sensory analysis, GC-MS

## Abstract

An untargeted gas chromatography-mass spectrometry (GC-MS) approach combined with sensory analysis was used to present the effects of different extraction methods (i.e., Pure Brew, V60, AeroPress, and French Press) on specialty graded *Coffea arabica* from Kenya. Partial Least Square Discriminant analysis and hierarchical clustering were applied as multivariate statistical tools in data analysis. The results showed good discrimination and a clear clustering of the groups of samples based on their volatile profiles. Similarities were found related to the filter material and shape used for the extraction. Samples extracted with paper filters (V60 and AeroPress) resulted in higher percentages of caramel-, and flowery-related compounds, while from metal filter samples (Pure Brew and French Press), more fruity and roasted coffees were obtained. Discriminant analysis allowed the identification of eight compounds with a high VIP (variable important in projection) discriminant value (i.e., >1), with 2-furanmethanol being the main feature in discrimination. Sensorial analyses were carried out through an expert panel test. The main evaluations revealed the French Press system as the lowest-scored sample in all the evaluated parameters, except for acidity, where its score was similar to V60. In conclusion, the data obtained from GC-MS analyses were in line with the sensorial results, confirming that the extraction process plays a fundamental role in the flavor profile of filter coffee beverages.

## 1. Introduction

Currently, approximately two billion cups of coffee are consumed daily worldwide [1,2]. Coffee beans are obtained from the two commercially available tropical species of the Rubiaceae family: *Coffea arabica* and *C. canephora*. The two species, which cover 70–80% and 30–40% [3,4] of the commercial market, respectively, differ in their geographical origin, climatic conditions, and altitude [5]. Arabica grows well at medium/high altitudes (1000 to 2100 m) with daily average temperatures of around 18 to 22 °C, typical of equatorial regions. This variety owns a most distinctive, tasteful, and intense aroma that results in a clear preference by consumers [6]. In contrast, Robusta coffee, which is less vulnerable to pests and diseases, is less requested than Arabica cultivars, although it has better resistance to hot and humid climates [6,7]. The flavor, which characterizes the confluence of aroma and taste, is a unique organoleptic attribute of coffee beverages [1,8]. Additionally, numerous factors, for example, harvest and post-harvest processing and the brewing method employed, characterized the chemical composition of brewed coffee [1,3,9]. As coffee consumption has increased worldwide, there has been growing interest in the flavor component, which plays an important role in marketing the product. The evaluation of coffee flavor and aroma is a fundamental step in the entire coffee production chain, from the selection of the raw material to the creation of the blends. In the coffee industry, sensory properties are measured using the cupping method. Although taste is a highly subjective matter, different tasters seem to have different opinions on the quality and value of a particular cup. In 1984, the Specialty Coffee Association of America (SCAA) proposed a detailed and standard protocol, adaptable to producers’ standardization needs, for the definition of the sensory quality of coffee [10,11]. The *Coffee Cupper’s Handbook* (1984) has helped to transform the work of cupping, based on the experience of the taster, into a science to maximize the level of standardization of the method [12,13]. In this analysis, panelists use their gustatory sensibilities to assign a score to sensory attributes (i.e., fragrance, flavor, aftertaste, acidity, body, balance, sweetness, and uniformity) [10]. In recent years, different extraction techniques have entered the market to create a lot of coffee-based beverages [5,14,15]. Recently, several brewing methods have been used to obtain coffee beverages. Among them, V60, French Press, and AeroPress are the most common extraction methods for the preparation of filter coffee or long coffee [16]. In fact, V60 is the traditional pour-over system, in which hot water is poured through coffee grounds in a filter paper; French Press is the classical full immersion system, with mechanical filtration; while AeroPress uses pressure, which is appropriate for strong extractions, and uses a paper filter [16,17,18]. Recently, an automated system called Pure Brew was introduced by Victoria Arduino [16]. It allows filter coffee preparation using an espresso coffee machine. To give a comprehensive overview of the filter coffee world, it was chosen to compare the four extraction methods mentioned above. They may differ in the grain size of ground coffee, extraction time, and amount of coffee and water used in the process [2]. In recent years, several analytical strategies have been implemented regarding the quality and integrity of foods, including coffee and coffee beverages, such as isotope ratio mass spectrometry (IRMS), liquid chromatography coupled with mass spectrometry (LC-MS), gas chromatography coupled with mass spectrometry (GC-MS), near infrared spectroscopy (NIRS), and nuclear magnetic resonance spectroscopy (NMR) [17,19]. However, to the best of our knowledge, there is still a lack of information in the scientific literature about the linkage between the volatile profiles and sensory traits of filter coffees and the related extraction methods. Since then [1,20,21] have deepened this concept, but using different approaches, extraction methodologies, and coffee compared with this study. Therefore, this research aimed to explore the potential correlations existing between the volatile profiles of specialty coffee beverages obtained through four extraction methods (Pure Brew, V60, AeroPress, and French Press) and their sensory profiles. This information is relevant to unraveling the effect of extraction method and coffee species combinations in terms of both sensory and chemical profiles.

## 2. Materials and Methods

### 2.1. Filter Coffee Sample Extraction Methods

One coffee variety was used for all analyses: *C. arabica* from Gardelli Specialty Coffee, Kakindu natural, Kenya, with medium roast, terroir Kiambu (Kenya), from variety “arabica” cultivar SL34, cultivated at 1800–2000 m a.s.l. Coffee was produced by small farmers, processed using the natural method, and dried on raised African beds. This type of coffee has a quality score of 89.00. Moreover, the beans were roasted with a customized solid-drum roaster. The package of coffee beans (250 g) was opened immediately before serving to avoid oxidative damage. The beans were ground with a professional grinder (Mythos one, Victoria Arduino, Belforte del Chienti, Italy) before each extraction. The grind was measured using the Mastersizer 3000 Aero Series dry disperser (Malvern PANalytical Ltd., Grovewood, UK): the grind was 1030 ± 2.13 μm for Pure Brew (PB), 1290 ± 3.81 μm for French Press (FP), 800 ± 3.21 μm for AeroPress (AP), and 945 ± 2.81 μm for V60. All samples were prepared with commercial natural water (Nerea). This water was selected for its mineral salt content (161 mg/L dry residue) associated with its salt balance. The chemical composition of the water was indeed suitable to be an effective extractant; according to the SCA parameters, the water must have a dry residue in the range of 75–250 mg/L and an ideal level of 100–150 mg/L [10,11]. This range ensures that the water contains enough minerals for proper extraction without overwhelming the coffee with excessive minerals.

The presence of minerals in the water was assessed by the producer (Nerea, Castelsantangelo sul Nera, MC, Italy). Magnesium concentration was 0.9 mg/L, and sodium concentration was 1.18 mg/L. Sodium should ideally be below 10 mg/L. Excessive sodium can lead to a salty taste. Regarding water hardness, it refers to the concentration of calcium ions. In coffee preparation, extremely soft water can result in under-extraction and flat flavors, while very hard water might lead to over-extraction and bitterness, so moderately hard water is generally considered ideal for coffee brewing. The SCA suggests a calcium hardness level of 50–175 ppm. The water used in this study reported a calcium concentration of 58 mg/L. Water pH is also an important parameter in coffee extraction. Water that is too alkaline or too acidic can impact the extraction process and alter the coffee’s flavors. The recommended pH range for brewing water is 6.5 to 7.5. The water used in this study had a pH of 7.45.

Each filter coffee extraction was replicated to obtain six samples of each method. The specific procedures used to obtain the four filter coffee samples are described in the following sections.

#### 2.1.1. Pure Brew

Pure-brew coffee was obtained with the VA388 Black Eagle Maverick machine (Simonelli Group, Victoria Arduino). Pure Brew technology is an extraction method that uses pulsating frequencies with low-pressure water (less than 0.15 bar). The Pure Brew filter consists of a micro-thin double-mesh conical basket that can contain up to 20 g of coffee. By combining Pure Brew technology with the patented filter basket, it was possible to obtain an automated single cup of filtered coffee. The water temperature was 93 °C. The coffee/water ratio was 1:16.6; the coffee/water ratio was 60 g/L.

#### 2.1.2. V60

Hario V60 is a patented system of the Japanese company Hario (Tokyo, Japan), consisting of a “V-shaped” coffee maker (with an angle of 60 °C, from which it takes its name). It consists of three parts: a carafe or glass base (Hario V60 Range Server, 600 mL), a ceramic drip coffee carafe with an inverted cone shape, and a paper filter (Hario V60 Paper Filter). First, a small amount of 93 °C hot water was poured to moisten the filter, and then the coffee was poured in until an apartment surface was formed. Next, 60 mL of 93 °C water was poured over the coffee, which had been pre-soaked for 15 s (the water was always poured out in concentric circles, starting in the center and then expanding to ensure a constant flow). After 30 s, another 100 mL of water was poured; finally, 130 mL of water was added at 1′20′. Finally, all the coffee was spun into the circuit, and the top of the unit was manually shaken three times. The final coffee to water ratio was 1:15, and 290 mL were yielded due to the coffee water retention ratio of approximately 2.1.

#### 2.1.3. AeroPress

AeroPress is a system invented in 2005 by Alan Adler as a manual coffee extraction machine that uses pressure generated by hand during the brewing process. The device consists of two nested cylinders, a chamber, and a piston with a hermetic seal. First, the paper filter (AeroPress^®^ Micro Filter, Palo Alto, CA, USA) was moistened, resting in a plastic filter holder attached to the syringe base, and then 15 g of coffee was added to the filter paper. Then, during the “blooming” phase, 60 mL of water was poured over the coffee bed to saturate all the coffee grounds. After pouring 60 mL of water, the coffee grounds had 15 s to fully saturate and release carbon dioxide. The water was topped up to a total of 290 mL, then stirred with a spatula. After another 20 s, the top of the AeroPress was pressed down with the hermetic seal, applying pressure for about 30 s. The final coffee to water ratio was 1:15, and 290 mL were yielded due to the coffee water retention ratio of approximately 2.1.

#### 2.1.4. French Press

The French Press (Lacor French Press wood) consists of a glass jug surrounded by a support with a handle and a plunger that passes through the lid and ends in a metal filter consisting of a fine mesh filter held between a spiral and a cross plate. At the beginning, the coffee was ground and put into the glass pot. Then, 93 °C hot water was added to 290 mL. During this process, turbulence was created from above by stirring at 1 m, 2 m, and 3 m; then, the lid of the device was removed and rotated four times with a spatula. At 4 m, the filter was slowly pushed into the coffee liquid to its full length. The ratio of coffee to water was 1:14.

### 2.2. Volatile Profiling through HS-SPME/GC Mass Spectrometry Analysis

A gas chromatography/mass selective detector (GC/MSD with PAL3) was used (Agilent, Santa Clara, CA, USA; Agilent 7890B GC Hardware with Agilent 5977 Series MSD; and MassHunter GC/MSD Data Aquisition (PAL3-Auto Sampler System) (MSD ChemStation software (Agilent, Version G1701DA D.01.00). The column used for separation was DB-WAX (0.25 mm × 60 m, 0.25 μm) (Agilent 122-7062, Santa Clara, CA, USA). The GC-MS workstation was an AgilentChem. The flow rate (He) was 1.2 mL/min in spitless mode. The injector temperature was 260 °C. The column temperature was programmed as follows: from 35 °C (4 min) to 120 °C (2.5 °C per min), from 120 °C to 250 °C (15 °C per min), then 250 °C for 3.33 min; the total run time was 50 min. Data were collected in electron impact (EI) mode and in SCAN mode, according to the previously published method [16]. In brief, the sample injection techniques with SPME were implemented through the PAL3 autosampler system. The fiber assembly was from Supelco (Bellefonte, PA, USA) and had a 50/30 μm coating of divinylbenzene/carboxy/polydimethylsiloxane (DVB/CAR/PDMS). For the analysis, 3 mL of each filtered coffee sample was placed in a shaker, where it was incubated at 60 °C and shaken at 250 rpm for 20 min. Then, the SPME was automatically injected into the gas-chromatographic system after adsorption. A desorption time of 10 min was sufficient to desorb the analytes from the fiber. Cleaning was performed automatically with the PAL system by inserting the fiber into the conditioning port at 230 °C for 20 min after each process.

### 2.3. Sensorial Analysis

Sensory analysis was performed by four qualified and certified panelists, two men and two women, in the age range of 34 to 65. All experiments were performed using the SCA cupping form [10,11,22]. In this method, accredited panelists assess sensory attributes: the aroma of the extracted coffee beverage; the taste perception of the coffee infusion, resulting from the interaction between aroma and taste; the aftertaste of the coffee infusion, resulting from the residual taste sensation on the back of the tongue; the acidity; and the mouthfeel of the infusion [23]. The assessors rated the quality of each global attribute on a scale of 6 (good) to 10 (outstanding) (Table 1). The protocol describes a temporal evaluation of sensory attributes.

The procedure is described below: First, the aroma is evaluated based on the inhalation of the brewed coffee while it is hot, as soon as it is served. Subsequent evaluation of flavor and aftertaste while the brewed coffee is hot, ensuring organoleptic and gustatory stimulation. Subsequently, the acidity, mouthfeel, and balance are assessed in a similar manner; the beverage is now cooler than originally served, which highlights different attributes of the brewed coffee. Finally, the overall score is based on the evaluation of all combined attributes with the brewed coffee at a temperature in the range of 30–45 °C [19].

### 2.4. Statistical Analysis

The VOCs database was uploaded to the MetaboAnalyst 5.0 tool (https://www.metaboanalyst.ca/ accessed on 25 March 2023) for statistical analyses. Missing values were replaced by LoDs (1/5 of the minimum positive value of each variable). The analysis was performed without normalizing the database. Therefore, Partial Least Squares-Discriminant Analysis (PLS-DA) was performed to evaluate the discrimination between the groups of samples. Thus, a hierarchical clustering analysis was performed and expressed using a heatmap to provide intuitive visualization of a data table.

The one-way analysis of variance (ANOVA) was applied to study the statistical significance of the values emerging from the sensorial analysis.

## 3. Results and Discussion

### 3.1. Effect of Different Brewing Methods on VOC Composition by HS-SPME/GC Mass Spectrometry Analysis

The volatile profiles of the six replicates of four filter coffee brewing methods were analyzed using the previously described method (Section 2.2) in full scan mode. The relative peak area percentage (RPA) of each scanned ion was calculated, and a total of 49 volatile compounds were identified (Table 2), representing 80.98 to 86.99% of the total headspace composition. The chemical classes detected were aldehydes (8), ketones (5), furans (11), phenolic compounds (3), pyridines (3), pyrazines (11), acids (2), terpene alcohols (3), and pyrroles (3). In this study, some molecules were found to have a very high RPA value, as already confirmed in the previous study [16]. Some of them are characterized by their capacity to stimulate odor receptors, also called “key odorants”, and they are highlighted in Table 2 with a “K”.

Furans and pyrazines are quantitatively highly abundant in coffee, as they are produced during the roasting process. Furans promote the characteristic malty, sweet, and roasted flavors with a high sensory threshold. This class is formed due to the Maillard reaction between amino acids or proteins and reducing sugars during the thermal degradation of carbohydrates, the oxidation of polyunsaturated fatty acids, and the degradation of ascorbic acid or its derivatives [24,25]. The highest 2-furanmethanol RPA values (9.45 ± 0.45%) [26] were found in PB (9.54 ± 0.45%). This molecule is related to candied, burnt, and smoky descriptors. Another important furan derivative is 2-furan methanol acetate, mainly related to fruity and banana flavors, which presented the highest RPA (7.90 ± 0.21%) in V60 samples.

Despite their low sensory threshold, pyrazines are potent aroma compounds that contribute to the characteristic coffee flavor. Pyrazines exhibit a nutty, earthy, roasted, and green aroma produced during the Maillard reaction [25,27]. Among pyrazines, methylpyrazine was the most prevalent. It showed an RPA range between 2.16 ± 0.11% in FP samples and 2.91 ± 0.06% in PB filter coffees. These molecules contribute to a chocolaty, corn-like, and nutty aroma in coffee cups [28]. 2,5-Dimethylpyrazine was the second-most abundant pyrazine in the analyzed samples. It was reported to be related to a nutty, peanut, musty, earthy, powdery, and slightly roasted cocoa powder nuance [5]. This compound was found with an RPA of 2.77 ± 0.25% in PB samples, resulting in the richest group. A similar trend was reported for 2,6-dimethylpyrazine, related to a cocoa and roasted nuts descriptor. The highest amount was found in PB and FP filter coffees (2.23 ± 0.02% and 2.05 ± 0.02%, respectively), while it was not detected in V60 or AP samples.

Aldehydes compounds are extender degradation and autoxidation products of unsaturated fatty acids, which are mainly responsible for the malty coffee flavor. Among these molecules, 2-methylpropanal, 2-methylbutanal, and 3-methylbutanal are classified as coffee key odorants as they have a major influence on the taste of coffee. The first was reported to have the higher RPA in V60 samples with a value of 0.42 ± 0.12%, while the others were mainly detected in PB and FP (1.11 ± 0.16% and 1.08 ± 0.29%, respectively, of 2-methylbutanal and 0.98 ± 0.12% and 1.32 ± 0.27%, respectively, of 3-methylbutanal). In addition, 5-methyl-2-furan carboxaldehyde, a furan derivative associated with high olfactometric activity [29] and with almond, caramel, sweet, and cooking attributes, was found at the highest concentration in V60, representing 10.38 ± 1.11% of the total area. The most detected compound of the aldehyde class was furfural, with a range between 16.46 ± 1.48% in FP and 19.51 ± 1.41% in V60 samples. Thus, furfural derivatives can be formed by the reaction between monosaccharides and an amino acid at high temperatures. The strong presence of furfural in the analyzed samples was reported to contribute to the cereal- and bread-like aroma in coffee [14].

Moreover, the pyrolysis of carbohydrates produces ketones that impart mushroom-like and even caramelized sweet notes [27]. Among ketones, 2,3-butanedione and 2,3-pentanedione are classified as key odorants, and they can be claimed to be associated with a buttery and creamy taste [7]. The highest RPA values of 2,3-butanedione were found in FP samples (0.31 ± 0.15%), while those of 2,3-pentanedione emerged in PB samples (0.94 ± 0.03%). A previous study concluded that ketones are particularly concentrated in filtered coffee infusions when compared with espresso coffee beverages [30].

Pyrroles, usually associated with nutty, hay-like, and herbaceous flavors [31], have also been detected in the four groups of analyzed samples. Their highest levels were reported in PB and AP samples. The FP coffee filter had the lowest level.

Among the phenolic compounds derived from thermal degradation of CQAs and characterized by spicy phenolic aroma and vanillin note, guaiacol and 4-vinyl guaiacol were the principal volatiles, being also coffee-key odorants [32]. The 4-vinyl guaiacol, known to be associated with spice and clove aromas, reported similar RPA within PB, V60, and FP samples (1.8 ± 0.11%, 1.58 ± 0.17%, and 1.62 ± 0.29%, respectively) and higher values in AP (2.76 ± 0.77%). In contrast, guaiacol was detected only in PB and FP (0.44 ± 0.13% and 0.24 ± 0.08%, respectively). Guaiacol is associated with sweet and medicinal flavors and evokes a burning sensation even at very low concentrations [33].

Pyridine is known to have fishy, roasted, and astringent properties, and can elicit a pungent, burnt taste even at concentrations in the mg/kg range. In coffee samples, pyridine is formed by degradation of trigonelline during the Maillard reactions activated due to the roasting processes [31]. This compound was mostly detected in PB samples (1.49 ± 0.11%). Among terpene alcohols, linalool and linalool oxides, *cis*-linalool oxide (furan) and *trans*-linalool oxide (furanoid), were detected. Linalool is related to flowery aromatic notes and was found similarly in all analyzed samples, with a range between 0.44 ± 0.12% and 0.59 ± 0.1% in FP and AP, respectively. The *cis*-linalool oxide (furan) that was reported to confer sweet-fruity aromatic notes to coffee [34] was mostly abundant in V60 samples (0.44 ± 0.12%).

In summary, volatile organic compounds (VOCs) are the most quality-determining constituents of coffee, occurring in concentrations ranging from parts per trillion (ppt) to parts per million (ppm). Among the 49 VOC compounds analyzed in this study, 10 are known as key aroma compounds (key odorants), as they can affect coffee taste and produce perceptible odors. These molecules can influence the aroma of the filter coffee cup when present in concentrations that exceed their odor threshold [25,28]. These odorants are of great importance because their loss or a slight change in their concentration can lead to coffee aroma or off-flavor defects [35].

### 3.2. Multivariate Statistical Discrimination of the Different Extraction Methods

**Figure 1:**
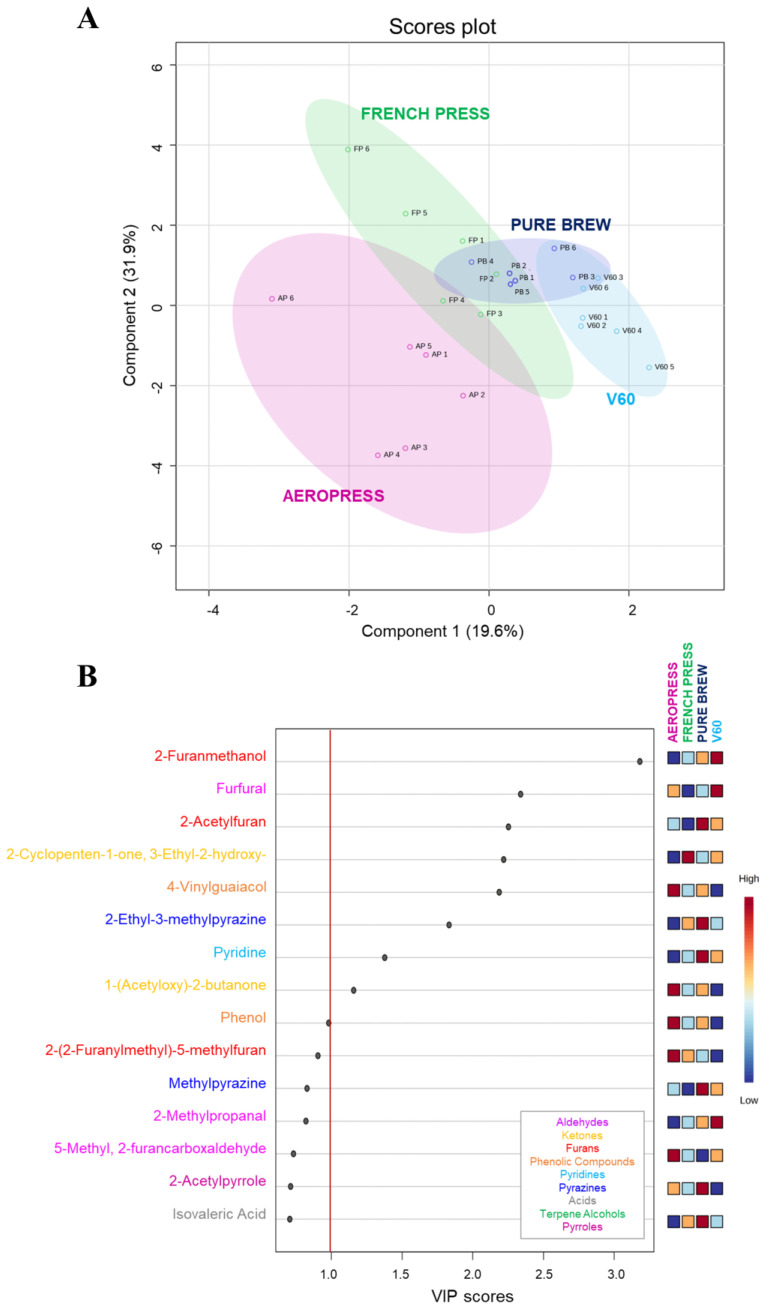
Discrimination of filter coffee samples according to extraction method based on VOC quantification results. (**A**) Partial least squares-discriminant analysis (PLS-DA) score plot. (**B**) Variable Importance in Projection (VIP) plot values of the volatile compounds, colored according to the chemical class. Red line indicates the relevant variable cut-off (VIP score > 1).

The VOC analysis of the four groups of filter coffee allowed the quantification of a total of 49 compounds, previously reported in Table 2. A chemometric tool has been applied to comprehensively assess if it is possible to discriminate the filter coffee extraction method from the aroma profile of the final product. The Partial Least Squared-Discriminant Analysis (PLS-DA) revealed discrimination in this sense (Figure 1A). A good grouping was obtained among the samples from the same group, underscoring the reproducibility of the extraction and the fact that each method allows for the extraction of a coffee with a specific aromatic profile. The FP and AP samples were farther apart than the replicates of the other two groups, revealing lower reproducibility of the extraction method. Manual turbulence as it is applied in FP and AP also has lower reproducibility where the coffee preparation processes involve a stirring/applied pressure step, but not in PB and V60. This parameter, together with the pressure, could be indicated as the main causes of the greater dispersion of AP and FP samples in the graph and therefore the lower reproducibility of these extraction methods, as they are strictly related to the operator’s skills. The PB coffee samples, obtained using an espresso coffee machine, and the V60 ones, obtained using a pour-over method exploiting the atmospheric pressure, are not so much affected by the operator. Moreover, PB and V60 extractions share the same conical filter shape, while in FP and AP extractions, rounded filters are used. The obtained results led us to assess that the filter shape can influence the volatile profile of filter coffee samples more than the filter material. Indeed, AP and V60 extractions, which involve a paper filter, resulted in coffee samples clearly discriminated based on the volatile profile. At the same time, a good volatile profile similarity was found between FP and PB samples, which were extracted using metal filters.

In the PLS-DA model, scores of VIP estimated the importance of each variable in the PLS projection [36]. Figure 1B represents the top 15 compounds with higher VIP scores, which fluctuated wildly among samples. In particular, nine of them reported a VIP score higher than 1.0, meaning they were important factors for the discrimination of filter coffee extraction methods based on the volatile profile. These VOCs belong to six different chemical classes: furans, aldehydes, ketones, pyrazines, pyridines, and phenolic compounds. The main factor influencing VIP was 2-furanmethanol, followed by furfural and 2-acetylfuran. These molecules are associated with high olfactometric activity [29] and with candy, burnt, caramel, and sweet attributes. V60 samples reported a higher concentration of the first two compounds, while the third was mainly present in Pure Brew samples, followed by V60.

**Figure 2:**
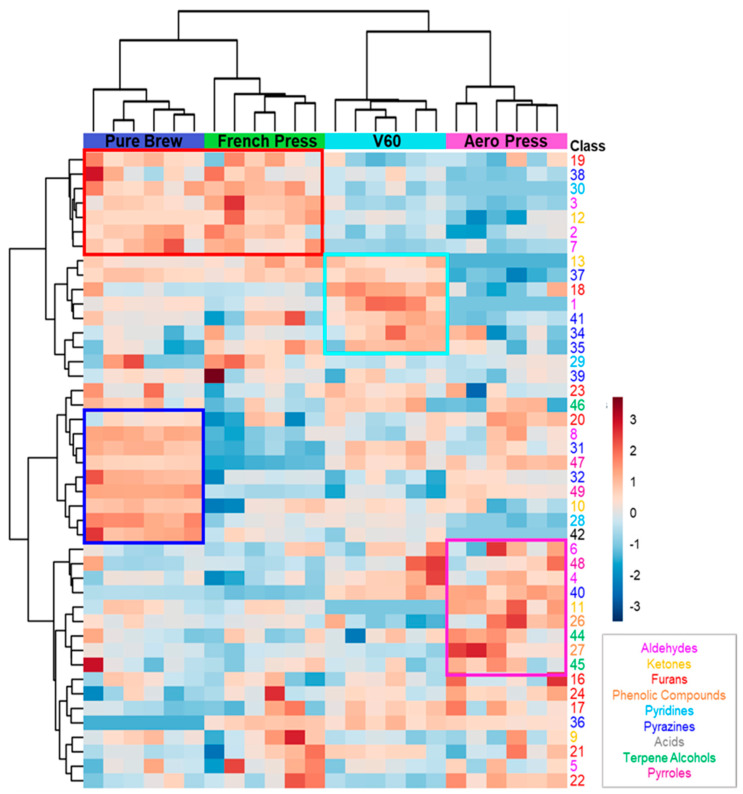
Heatmap of VOCs analyzed by hierarchical clustering. Each row represents a single compound, which is colored according to the chemical class. Each column represents an individual filter coffee sample. Cluster analysis was performed using Ward method. Red indicates a high content, and blue indicates a low content of each VOC.

The possibility to discriminate the extraction method of the analyzed samples based on their volatile profile was confirmed by the application of hierarchical clustering, and the results are shown in a heatmap in Figure 2. Good clustering was obtained among the four groups. First, clustering between PB and FP on the one hand and V60 and AP on the other was obtained. This was mainly due to the detected concentrations of the first seven features (red squared in the figure). These molecules were more present in filter coffees obtained from metal filter extractions. Analyzing the aromatic notes of this short list of VOCs, it can be seen that they are mainly related to malty, fruity, and nutty aromas. The following group of seven features characterize the V60 filter coffee samples, and they were mainly associated with sweet, earthy, and buttery oil aromatic notes (light blue squared). The next ten VOCs were highly concentrated in PB samples, resulting in roasted coffee, nutty, corn-like, and chocolate aromas (blue squared). A similar trend for the purple squared region of the heatmap is related to features mainly detected in AP samples. These VOCs confer caramel, flowery, and spicy aromatic notes to AP filter coffees.

This analysis identifies the volatile compounds that characterize the different filter coffees studied and give them specific sensory characteristics. Therefore, it was noted that the beverages obtained with the metal filter have a different volatile profile from those obtained with the paper filter. Furthermore, within the two groups, the shape of the filter also plays a fundamental role. In conclusion, analyzed coffee samples could be discriminated according to their preparation technique using the statistical tools of multivariate analysis.

### 3.3. Sensory Analysis

**Figure 3:**
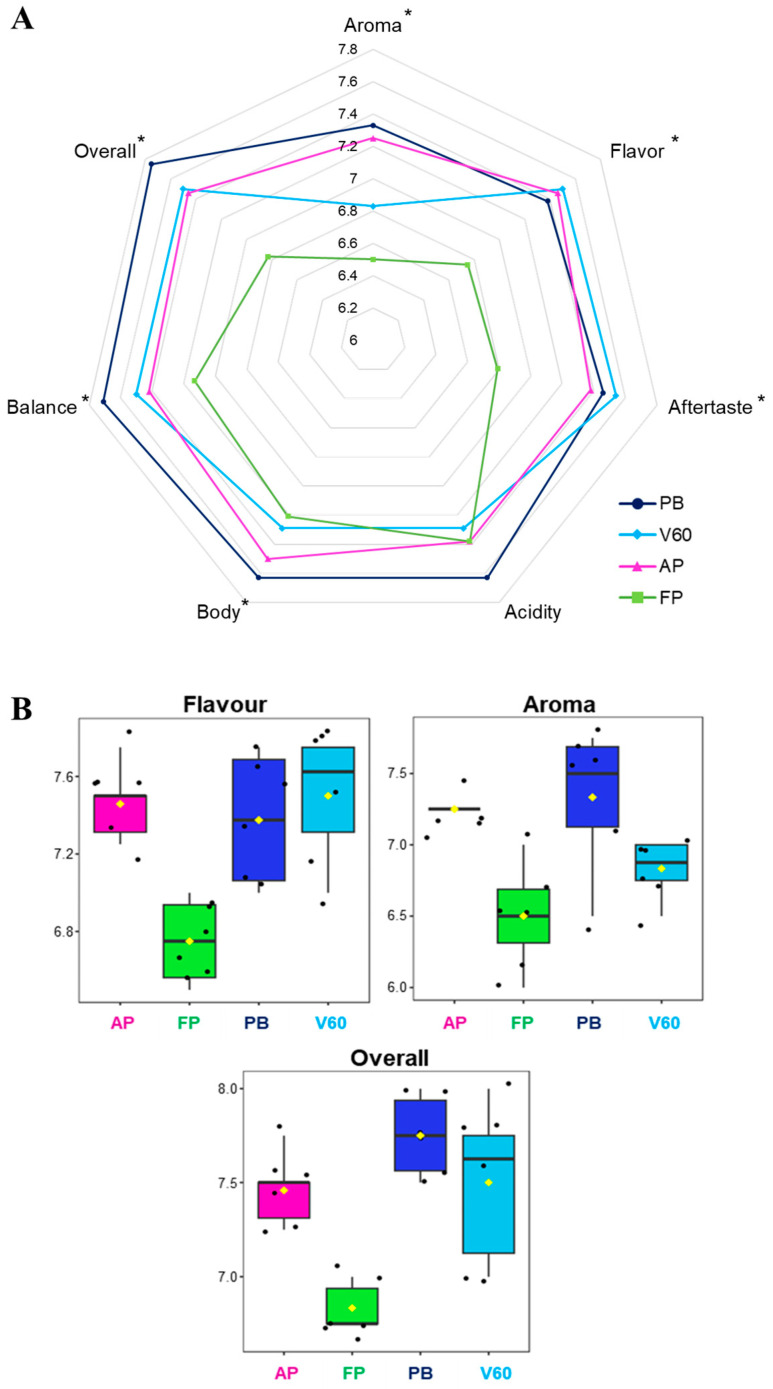
Sensorial analysis results of the four filter coffee comparisons. (**A**) Spider gram with the attributed values. Statistically different values according to ANOVA results are reported with an asterisk. (**B**) Feature view plots of the three main sensorial parameters related to coffee volatile profile extracted from the ANOVA loadings plot.

The SCA (Specialty Coffee Association) protocol is designed for quality assessment, and finding significant differences is complex because the protocol is based on a specific description of sensory attributes [37]. Aroma, flavor, aftertaste, acidity, body, balance, and an overall score were the evaluated parameters. Sensorial analysis results are represented in a spider graph in Figure 3A, while the adopted quality scale and the values can be consulted in Table 1 and Table 3. A statistically significant difference was reported for all the analyzed parameters between the four groups of samples except for acidity. In general, PB filter coffees were valued with the highest scores of aroma, body, balance, acidity, and overall impression, while FP reported the lowest scores of all the investigated aspects except for acidity, which was similar to the others. The chosen specialty coffee from Kenya is well-known for its flavor, complexity, and acidity notes. Coffee cup acidity derives from several factors, not only the extraction method. In this study, this parameter was evaluated through a sensorial analysis to compare the beverages from different extraction methods, reporting all similar (no statistically significant differences) and elevated scores due to this intrinsic characteristic of coffee.

A consistent sense of sweetness and roundness was observed in the PB samples, with a smoother and fuller acidity; the acidity ranged from 7.50 to 7.75 (the highest acidity score). In addition, the aftertaste was smoother and less sweet than the other extraction methods used for this analysis (average value of 7.46).

For V60, however, acidity predominated (the average acidity score was 7.75); in this case, roundness was lacking (probably due to the use of the paper filter). This lack of roundness could be related to the high presence of pyrazines in this system, such as 2-ethyl-3-methylpyrazine, 2,3-dimethylpyrazine, and 2-ethyl-6-methylpyrazine, which are associated with earthiness and mustiness in the literature.

Meanwhile, AeroPress extractions gave a flat result in the cup, with little body and excess coffee grounds in the middle of the results, despite the higher presence of molecules associated with positive notes, such as the class of terpene alcohols. Given the concentrations and the method of extraction, the final result was a watery cup of coffee.

Finally, the French press cups, on average, gave a higher result than the AeroPress cups. In summary, the differences between the extraction methods resulted in diverse cups (one more watery, one sweeter, and another more intense). The result that unites the Pure Brew and French Press systems is related to the presence of two aldehydes most detected in both systems: 2-methylbutanal and 3-methylbutanal, which are associated with fruity and nutty flavors, and both share the metal filter. In conclusion, from the sensorial analysis, Pure Brew resulted in the most performing extraction system as it was assigned the highest score in terms of overall 7.75 and also resulted in the most balanced, acidic, aromatic, and pleasant cup of filter coffee among the analyzed.

Figure 3B reports the feature view plots of the three main sensorial parameters related to the coffee volatile profile extracted from the ANOVA loadings plot. It is possible to appreciate that the statistical significance was mainly associated with low FP values. This data confirmed the results obtained in the VOCs analysis; in fact, aroma, flavor, and other sensorial characteristics are closely related to the presence of specific volatile analytes, as well as carbohydrates, lipids, organic acids, and chlorogenic acids.

## 4. Conclusions

The present study investigated the volatile profile and sensorial traits of *C. arabica* Kakindu natural, Kenya, medium roast specialty coffee prepared using four different extraction techniques, namely Pure Brew, V60, AeroPress, and French Press, to obtain filter coffee beverages. A GC-MS method was applied to analyze volatile organic compounds in the four groups of samples that were also subjected to sensorial analysis according to the SCA cupping form. The results of GC-MS were in line with the sensorial analysis, and their combination allowed for the establishment of characteristic profiles of the brewed coffees, revealing mutual differences and similarities. Comparing the coffee preparations, the filter attributes, materials, and shapes significantly impact the sensorial traits of the resulting filter coffees. The application of the PLS-DA statistical tool allowed the discrimination of filter coffee extraction methods based on volatile organic compounds. This approach confirmed that, using the same coffee material, each filter coffee extraction method results in a cup with a specific and characteristic aromatic profile. Further studies based on more targeted approaches are advisable to better assess the correlation between sensory perceptions and chemical markers as functions of the extraction technique.

## Figures and Tables

**Table 1:** Quality scale used for sensorial analysis.

6.00—Good	7.00—Very Good	8.00—Excellent	9.00—Outstanding
6.25	7.25	8.25	9.25
6.50	7.50	8.50	9.50
6.75	7.75	8.75	9.75

**Table 2:** Volatile compounds detected in different filter coffee extraction methods by HS-SPME/GC-MS and related aromatic notes. Values are reported as area % mean ± standard deviation of the six analyzed samples of each filter coffee extraction method. Key odorants are signed with a K on the left row.

		Compounds Name and Classes	Sens. Threshold (ppb)	Aromatic Notes	Pure Brew	V60	AeroPress	French Press
		Aldehydes						
1	K	2-Methylpropanal		Buttery oil	0.26 ± 0.02	0.42 ± 0.12	n.d. *	0.2 ± 0.05
2	K	2-Methylbutanal	1.3/1.9	Malty	1.11 ± 0.16	0.59 ± 0.16	0.5 ± 0.32	1.08 ± 0.29
3	K	3-Methylbutanal	0.35/0.4	Malty, Fruity, Almond, Aldehydic	0.98 ± 0.12	0.62 ± 0.17	0.44 ± 0.09	1.32 ± 0.27
4		Furfural		Sweet, Caramel	17.76 ± 0.8	19.51 ± 1.41	18.76 ± 1.03	16.46 ± 1.48
5		Benzaldehyde		Strong, Sharp, Sweet, Bitter, Almond, Cherry	0.65 ± 0.17	0.61 ± 0.1	0.69 ± 0.14	0.79 ± 0.3
6		5-Methyl, 2-furancarboxaldehyde	6000	Caramel	9.74 ± 0.39	10.38 ± 1.11	10.7 ± 1.52	9.89 ± 0.84
7		1-Methyl-1H-pyrrole-2-carboxaldehyde		Musty	1.78 ± 0.26	1.20 ± 0.04	1.26 ± 0.14	1.72 ± 0.19
8		5-Ethylfurfural			0.95 ± 0.04	0.58 ± 0.15	0.69 ± 0.2	0.39 ± 0.16
		SUM			32.97 ± 1.96	33.92 ± 3.26	33.04 ± 3.44	31.85 ± 3.58
		Ketones						
9	K	2,3-Butanedione	0.3/0.15	Buttery oil	0.28 ± 0.05	0.20 ± 0.03	0.25 ± 0.09	0.31 ± 0.15
10	K	2,3-Pentanedione	20/30	Buttery oil	0.94 ± 0.03	0.78 ± 0.07	0.62 ± 0.22	0.59 ± 0.37
11		1-(Acetyloxy)-2-butanone			0.38 ± 0.15	n.d.	0.64 ± 0.16	0.34 ± 0.12
12		1-Propanone, 1-(2-furanyl)-			1.07 ± 0.01	0.83 ± 0.07	0.55 ± 0.29	1.22 ± 0.2
13		2-Cyclopenten-1-one, 3-Ethyl-2-hydroxy-		Fruity, Caramel, Nutty	1.22 ± 0.13	1.44 ± 0.17	n.d.	1.52 ± 0.35
		SUM			4.49 ± 0.37	3.25 ± 0.34	2.06 ± 0.76	3.98 ± 1.19
		Furans						
14		2-Methylfuran		Ethereal, Acetone, Chocolate	n.d.	n.d.	0.71 ± 0.19	0.51 ± 0.33
15		2-(Methoxymethyl)furan			n.d.	n.d.	0.42 ± 0.22	n.d.
16		3(2H)-Furanone, dihydro-2-methyl-			0.5 ± 0.12	0.49 ± 0.11	0.61 ± 0.26	0.44 ± 0.2
17		2-n-Butyl furan			0.44 ± 0.1	0.71 ± 0.08	0.7 ± 0.2	0.56 ± 0.3
18		2-Furanmethanol acetate		Sweet, Fruity, Banana, Horseradish	6.84 ± 0.58	7.90 ± 0.21	6.38 ± 0.86	6.54 ± 0.24
19		2-Furanmethanol propanoate			0.77 ± 0.11	0.45 ± 0.14	0.52 ± 0.19	0.69 ± 0.22
20		2,2′-Methylenebisfuran		Roast	0.78 ± 0.1	0.70 ± 0.15	0.93 ± 0.15	0.49 ± 0.37
21		2-Furanmethanol		Candy, Burnt, Smoky	8.7 ± 0.26	9.54 ± 0.45	9.24 ± 1.19	9.28 ± 1.63
22		2-(2-Furanylmethyl)-5-methylfuran		Hearty, Mushroom	0.5 ± 0.14	0.4 ± 0.05	0.8 ± 0.11	0.7 ± 0.28
23		2-Acetylfuran			3.79 ± 0.97	3.73 ± 0.45	2.9 ± 1.4	2.84 ± 0.77
24		α-Furfuryliden-α-furylmethylamine			0.3 ± 0.14	0.43 ± 0.05	0.52 ± 0.08	0.45 ± 0.18
		SUM			22.62 ± 2.52	24.35 ± 1.69	23.73 ± 4.85	22.51 ± 4.52
		Phenolic Compounds						
25	K	Guaiacol	2.5	Phenolic, Burnt, Spicy	0.44 ± 0.13	n.d.	n.d.	0.24 ± 0.08
26		Phenol			0.57 ± 0.09	0.38 ± 0.31	0.84 ± 0.33	0.51 ± 0.08
27	K	4-Vinylguaiacol	0.75/20	Spicy, Woody	1.8 ± 0.11	1.58 ± 0.17	2.76 ± 0.77	1.62 ± 0.29
		SUM			2.81 ± 0.33	1.96 ± 0.48	0.8 ± 0.15	1.67 ± 0.44
		Pyridine						
28		Pyridine	77	Sour, Fishy, Amine	1.49 ± 0.11	0.89 ± 0.06	0.43 ± 0.09	0.77 ± 0.17
29		4(H)-Pyridine, N-acetyl-			0.38 ± 0.33	0.32 ± 0.07	0.37 ± 0.06	0.57 ± 0.19
30		2-Acetylpyridine			0.31 ± 0.09	0.08 ± 0.08	n.d.	0.33 ± 0.08
		SUM			2.18 ± 0.53	1.29 ± 0.21	0.8 ± 0.15	1.67 ± 0.44
		Pyrazine		Roasted odour of coffee				
31		Methylpyrazine		Chocolate, Corn-like, Nutty	2.91 ± 0.06	2.75 ± 0.17	2.57 ± 0.19	2.16 ± 0.11
32		2,5-Dimethylpyrazine	80	Musty, Earthy, Powdery and slightly roasted with Cocoa powder nuance	2.77 ± 0.25	1.81 ± 0.38	2.24 ± 0.13	1.89 ± 0.42
33		2,6-Dimethylpyrazine			2.23 ± 0.21	n.d.	n.d.	2.05 ± 0.02
34		2,3-Dimethylpyrazine	800	Musty, Nut skins, Cocoa powdery with potato and coffee nuances	0.32 ± 0.06	0.43 ± 0.08	0.33 ± 0.13	0.36 ± 0.06
35	K	2-Ethyl-6-methylpyrazine		Earthy, Musty	1.55 ± 0.17	1.81 ± 0.18	1.61 ± 0.16	1.82 ± 0.1
36		2-Ethyl-5-methylpyrazine			n.d.	1.57 ± 0.19	1.42 ± 0.08	1.59 ± 0.14
37		2-Ethyl-3-methylpyrazine			1.62 ± 0.09	1.56 ± 0.11	0.63 ± 0.21	1.58 ± 0.12
38		2,6-Diethylpyrazine			0.51 ± 0.21	0.39 ± 0.06	0.27 ± 0.06	0.51 ± 0.12
39	K	3-Ethyl-2,5-dimethylpyrazine		Fried, Peanut aroma and Chocolate	1.51 ± 0.04	1.47 ± 0.09	1.45 ± 0.06	1.56 ± 0.18
40		2-Ethyl-3,5-dimethylpyrazine			0.83 ± 0.01	1.62 ± 0.18	1.79 ± 0.19	0.55 ± 0.09
41	K	3,5-Diethyl-2-methylpyrazine		Earthy	0.63 ± 0.05	0.73 ± 0.05	0.48 ± 0.06	0.61 ± 0.24
		SUM			14.88 ± 1.15	14.14 ± 1.49	12.79 ± 1.27	14.68 ± 1.61
		Acids						
42		Isovaleric acid			0.67 ± 0.16	0.32 ± 0.06	n.d.	0.29 ± 0.1
43		Nonanoic acid			n.d.	0.35 ± 0.07	n.d.	0.18 ± 0.06
		SUM			0.67 ± 0.16	0.67 ± 0.13	n.d.	0.47 ± 0.16
		Terpene Alchols						
44		Linalool	0.17	Flowery	0.45 ± 0.11	0.45 ± 0.17	0.59 ± 0.11	0.44 ± 0.12
45		cis-Linalool oxide (furan)			0.38 ± 0.21	0.31 ± 0.08	0.44 ± 0.12	0.35 ± 0.09
46		trans-Linalool oxide (furanoid)			0.7 ± 0.11	0.67 ± 0.17	0.54 ± 0.25	0.48 ± 0.14
		SUM			1.53 ± 0.43	0.76 ± 0.42	1.57 ± 0.48	1.27 ± 0.35
		Pyrrole						
47		1-Furfurylpyrrole		Defective beans, negative notes	1.78 ± 0.04	1.63 ± 0.25	1.85 ± 0.29	0.31 ± 0.11
48		Pyrrole-2-aldehyde			1.27 ± 0.21	1.57 ± 0.33	1.46 ± 0.22	1.22 ± 0.07
49		2-Acetylpyrrole			1.79 ± 0.06	0.76 ± 0.35	1.27 ± 0.11	0.66 ± 0.06
		SUM			4.84 ± 0.31	3.96 ± 0.93	4.58 ± 0.62	2.19 ± 0.24
		TOTAL IDENTIFIED COMPOUNDS %			86.99 ± 7.76	84.3 ± 8.95	82.17 ± 12.67	80.98 ± 12.53

n.d. *: not detected (peak area value below 5 × 10^4^).

**Table 3:** Sensorial analysis results. Statistically significant differences between groups of samples are reported with different letters on mean values.

	SAMPLES	Aroma	Flavor	Aftertaste	Acidity	Body	Balance	Overall
PB 1	Pure Brew	6.5	7	7	7.5	7.25	7.5	7.75
PB 2		7.5	7	7.5	7.75	7.75	7.75	8
PB 3		7.75	7.75	7.75	7.75	8	8	8
PB 4		7	7.5	7.5	7.75	7.5	7.5	7.5
PB 5		7.5	7.75	7.5	7.5	7.75	7.75	7.75
PB 6		7.75	7.25	7.5	7.5	7.5	7.75	7.5
	Mean Values	7.33 ^a^	7.38 ^a^	7.46 ^a^	7.63	7.63 ^a^	7.71 ^a^	7.75 ^a^
V60 1	V60	6.75	7	7	6.75	7.25	6.75	7
V60 2		7	7.75	7.5	7	7	7.5	7.5
V60 3		7	7.75	8	7.75	7.5	7.75	8
V60 4		6.5	7.75	7.5	7.5	7.5	7.75	7.75
V60 5		7	7.25	7.5	7.5	7.5	7.5	7
V60 6		6.75	7.5	7.75	7.25	7	7.75	7.75
	Mean Values	6.83 ^a^	7.50 ^a^	7.54 ^a^	7.29	7.29 ^a,b^	7.50 ^a,b^	7.50 ^a^
AP 1	AeroPress	7.25	7.5	7.25	7	7.25	7.25	7.25
AP 2		7.25	7.5	7.25	7.25	7.5	7.25	7.5
AP 3		7.25	7.75	7.5	7.75	7.75	7.75	7.75
AP 4		7	7.25	7.5	7.5	7.5	7.5	7.5
AP 5		7.5	7.25	7.25	7.5	7.75	7.5	7.25
AP 6		7.25	7.5	7.5	7.25	7.25	7.25	7.5
	Mean Values	7.25 ^a^	7.46 ^a^	7.38 ^a^	7.38	7.50 ^a,b^	7.42 ^a,b^	7.46 ^a^
FP 1	French Press	6	6.5	6.5	7	7.25	7	6.75
FP 2		6.5	7	6.75	7.25	7.25	7	6.75
FP 3		6.75	7	7.25	7.75	7.5	7.5	6.75
FP 4		7	6.75	7	7.25	7	7.25	7
FP 5		6.25	6.75	6.75	7.5	7	7	7
FP 6		6.5	6.5	6.5	7.5	7.25	7	6.75
	Mean Values	6.50 ^b^	6.75 ^b^	6.79 ^b^	7.38	7.21 ^b^	7.13 ^b^	6.83 ^b^
	*p*.value	0.0009	0.0004	0.0003	0.1962	0.0209	0.0082	0.5666

## Data Availability

The data used to support the findings of this study can be made available by the corresponding author upon request.
